# Trehalose and bacterial virulence

**DOI:** 10.1080/21505594.2020.1809326

**Published:** 2020-08-30

**Authors:** Muthita Vanaporn, Richard W Titball

**Affiliations:** a Department of Microbiology and Immunology, Faculty of Tropical Medicine, Mahidol University, Bangkok, Thailand; b College of Life and Environmental Sciences, University of Exeter, Exeter, UK

**Keywords:** Trehalose, virulence, pathogen, bacteria

## Abstract

Trehalose is a disaccharide of two D-glucose molecules linked by a glycosidic linkage, which plays both structural and functional roles in bacteria. Trehalose can be synthesized and degraded by several pathways, and induction of trehalose biosynthesis is typically associated with exposure to abiotic stress. The ability of trehalose to protect against abiotic stress has been exploited to stabilize a range of bacterial vaccines. More recently, there has been interest in the role of this molecule in microbial virulence. There is now evidence that trehalose or trehalose derivatives play important roles in virulence of a diverse range of Gram-positive and Gram-negative pathogens of animals or plants. Trehalose and/or trehalose derivatives can play important roles in host colonization and growth in the host, and can modulate the interactions with host defense mechanisms. However, the roles are typically pathogen-specific. These findings suggest that trehalose metabolism may be a target for novel pathogen-specific rather than broad spectrum interventions.

## Introduction

Trehalose is a disaccharide of two D-glucose molecules linked by a glycosidic linkage. There are three possible structures of trehalose (α,β-1,1-, β,β-1,1-, and α,α1,1-) but the α,α-1,1-trehalose form is the predominant form found in living organisms[1]. The 1,1 linkage provides structural rigidity and consequently unique hydration properties [2]. Trehalose is produced by a wide variety of plants and algae, many invertebrates[1] and by microorganisms such as bacteria, yeasts, and fungi [3,4]. Mammals, including humans, do not have the ability to synthesis trehalose, though they can use exogenous trehalose as a carbon source.

Trehalose plays a wide range of functional and structural roles in organisms. In insects, trehalose is the major sugar in hemolymph, providing an instant source of energy, for example during flight [5]. Possibly the most dramatic evidence of trehalose-mediated resistance to stress is the desiccation response of the resurrection plant (*Selaginella lepidophylla*) [6]. Dried, and apparently dead plants, are able to recover when re-hydrated because of the ability of trehalose to protect molecules and cells from irreversible damage. What has become apparent more recently is that trehalose-6-phosphate can regulate metabolism, including the regulation of energy generating pathways and in plants the regulation of photosynthesis [7]. A previous review proposed that the loss of trehalose metabolism is associated with reduced pathogenicity [8], and there have been some important new findings in this field since 2013. This review considers the roles of trehalose in bacteria, and especially the interplay between trehalose biosynthesis, trehalose degradation, the regulation of metabolism and virulence, and the ways in which trehalose biology can be exploited to control disease.

### Trehalose biosynthesis pathways

**Figure 1.**
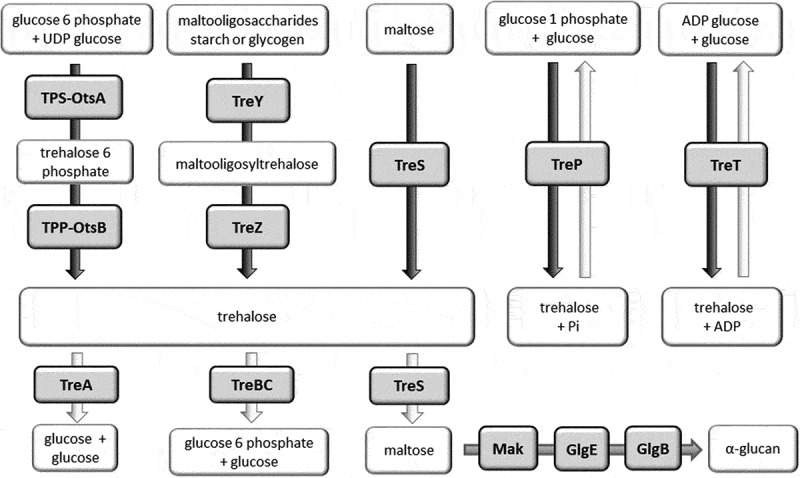
Trehalose biosynthesis (black arrows), trehalose degradation (white arrows) and α-glucan biosynthesis (gray arrow) pathways in bacteria. Enzymes are shown in gray shaded boxes.

Trehalose can be synthesized by several pathways, and some organisms possess more than one biosynthetic pathway, reflecting the importance of this disaccharide. The most common is the TPS/TPP (also known as OtsBA) biosynthetic pathway. This pathway involves two enzymes, trehalose-6-phosphate synthase (TPS or OtsA) and trehalose-6-phosphate phosphatase (TPP or OtsB), to convert glucose-6-phosphate and UDP-glucose to trehalose (Figure 1). An alternative TreYZ pathway involves the production of trehalose from maltooligosacharides by maltooligosyltrehalose synthase (TreY) and maltooligosyltrehalose trehalohydrolase (TreZ). The TreS biosynthetic pathway involves a single trehalose synthase (TreS) enzyme which is able to reversibly inter-convert trehalose and maltose. The TreP and TreT biosynthetic pathways are also reversible. The TreP pathway involves a trehalose phosphorylase enzyme (TreP). The TreT pathway generates trehalose from ADP-glucose and glucose using trehalose glycosyltransferring synthase enzyme (TreT). All of these trehalose synthesis pathways are found in bacteria [9].

In *E. coli* [10], expression of *otsBA* is linked to RpoS, the master stress-response regulator [10]. However, exposure to a range of stresses can induce trehalose biosynthesis in an RpoS-independent manner. Classically trehalose biosynthesis is induced by hyper-osmotic stress or entry of bacterial cultures into the stationary phase [11]. Cold-shock can also induce trehalose synthesis by the *otsBA* system [12]. Exposure to ethanol or NaCl stress can induce trehalose biosynthesis in *P. aeruginosa* via the TreYZ pathway [13].

### Trehalose import and degradative pathways

Alternative pathways exist for the import and degradation of trehalose by bacteria (Figure 1). Trehalose may enter the cell either via a permease and then be hydrolyzed by a trehalase. Alternatively, under low osmolarity conditions in *E. coli*, trehalose is converted to trehalose-6-phosphate which is then taken up by a trehalose-specific enzyme II (TreB) of the phosphotransferase system [10,14]. The trehalose-6-phosphate is subsequently hydrolyzed to glucose and glucose-6-phosphate by TreC. The encoding *treB* and *treC* genes form an operon (the *tre* operon) including *treR* which encodes a regulator of the operon [14]. The expression of *treB* and *treC* is induced by trehalose-6-phosphate and repressed by TreR [15].

The nomenclature of the genes involved in trehalose hydrolysis is rather confusing and gene names describing the biological activity and cellular locations of proteins involved are not used consistently. In *E. coli* there are at least three enzymes involved in trehalose hydrolysis. TreA is a trehalase found in the periplasmic space, hydrolyzing trehalose to glucose under conditions of high osmolarity [16,17], and the glucose generated can then be imported by the PTS [17]. An isoform of the TreA trehalase, termed TreF [17], is reported to be in the cytoplasmic compartment and also appears to play a key role in the degradation of trehalose synthesized within the bacterial cell under conditions of high osmolarity [16]. In some bacteria, these enzymes are called TreH, which is the nomenclature normally used for eukaryotic trehalases capable of hydrolyzing trehalose to glucose [18].

In the prototypic Gram-positive bacterium *Bacillus subtilis* trehalose-6-phosphate is hydrolyzed by TreA, a protein with sequence homology to the *E. coli* TreC enzyme [19]. TreA forms part of an operon with the TreP, an enzyme that phosphorylates of trehalose to trehalose-6-phosphate to import into the bacterial cell. The operon is repressed by TreR [20]. Overall, this operon in *B. subtilis* shows organizational, functional, and sequence similarity with the *tre* operon of *E. coli*.

Many other bacterial species possess an operon similar to the *tre* operons in *E. coli* and in *B. subtilis*, and expression of the genes within these operons is reported to be upregulated by trehalose [20–22]. However, there are important differences in the regulation of these operons. For example, *Streptococcus mutans* TreR activates the operon [21] whereas the corresponding proteins in *E. coli* and *B. subtilis* are repressors [15,20].

### Trehalose lipids

Trehalose lipids are found in the cell wall of some bacterial species, but are best studied in the Mycobacteria. In *M. tuberculosis* a range of glycolipids, which are derived from trehalose have been reported including sulfolipids [23,24], diacyltrehaloses, triacyltrehaloses and polyacyltrehaloses [24], trehalose 6,6 mycolate (TMM) and trehalose 6,6 dimycolate (TDM) and lipooligosacharides [24]. Trehalose is converted into TMM by a polyketide synthase (Pks13) and subsequently exported into the periplasm by mycobacterial membrane protein large 3 (MmpL3) [25]. The Ag85 complex (Ag85s) facilitates TDM biosynthesis from TMM [25]. Trehalose can also serve as a substrate in the GlgE pathway which converts trehalose into α-glucans using four enzymes TreS, Mak, GlgE, and GlgB [26]. This pathway is believed to play a role in the synthesis of cell wall components such as capsular α-glucan and methyl glucose lipopolysaccharides [24,25]. Many of these trehalose lipids, and the pathways using trehalose as a substrate, play roles in virulence [24,27]. They are targets for anti-mycobacterial drugs (see below).

**Figure 2.**
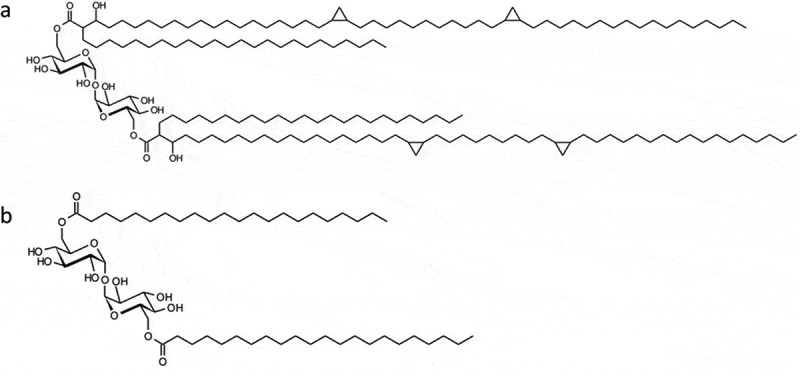
Structures of a) TDM (cord factor) and b) TDB, a low toxicity derivative of TDM which has been evaluated as a vaccine adjuvant.[62]

TDM (or cord factor) is the best known example of a trehalose lipid in *M. tuberculosis* [24]. Similar glycolipids are found in the cell walls of other *Mycobacteria, Nocardia,* and *Corynebacteria* species [28]. It is synthesized by the esterification of trehalose to two mycolic acid residues (Figure 2). The lengths of the fatty acid chains differ between species to species, but are typically 40–60 carbons in *Nocardia* and *Corynebacteria* and 70–90 carbons in the *Mycobacteria* [29,30]. The presence of TDM in the cell wall promotes the inhibition of fusion between bacteria and phospholipid vesicles, such as phagosomes and lysosomes, in the host cell [29]. TDM is also partly responsible for the low permeability of the mycobacterial cell wall, which consequently confers drug resistance [30]. Recently, the trehalose phospholipids 6,6,-diphosphatidyltrehalose (diPT) and 6-phosphatidyltrehalose (PT) have been discovered in a group of related gram-negative bacteria which includes some *Salmonella* and *Escherichia*isolates [31]. Like TDM, diPT is a symmetrical molecule with a trehalose core and lipids attached at the 6-positions, and it is a ligand for macrophage inducible Ca2-dependent lectin receptor (Mincle) [31]. However, the roles of diPT and PT in disease are yet to be determined.

### Role of trehalose in protection against stress

Trehalose can play a key role in protecting bacteria, against a range of stresses. One of the earliest known examples is the observation that increasing levels of trehalose in spores of *Streptomyces griseus* provide increasing levels of resistance to heat and dessication stress [32], and this likely contributes to the ability of actinomycete spores to withstand harsh environmental conditions. High levels of accumulated trehalose also delayed the germination of spores, though the significance of this is not clear. Since then, trehalose has been shown to also protect bacterial vegetative cells from a range of abiotic stresses. An *E. coli* mutant unable to produce trehalose survived poorly at 4°C compared to wild type bacteria [12]. Transformation with the *otsA/otsB* genes restored the ability of the bacteria to synthesize trehalose and resistance to cold stress [12]. Resistance to heat stress has been demonstrated in a number of bacteria and trehalose biosynthesis (*ots*) mutants of *Acinetobacter baumanii* [33] *E.coli* [34], *Salmonella enterica* [35], and *Rhizobium etli* [36] showed increased susceptibility to heat stress. Conversely, trehalose degradation (*treA*) mutants of*Listeria monocytogenes* [37] and *Burkholderia pseudomallei* [38] showed increased resistance to heat (and other) stress, as a consequence of the elevated levels of trehalose within bacterial cells. Endogenously produced trehalose can also protect a range of bacterial vegetative cells against desiccation stress [11,39,40]. TDM might perform a similar function in *M. tuberculosis* by protecting membranes from damage caused by desiccation [41]. Trehalose is rarely reported to protect bacteria against oxidative stress, even though this is a commonly reported function in fungi [4]. Disruption of trehalose biosynthesis in *Xanthomonas citri* reduced resistance of the bacteria to H_2_O_2_ oxidative stress [42]. However, in many bacteria inactivation of trehalose biosynthesis pathways did not decrease resistance to oxidative stress (Table 1). Rather, the reverse situation has been reported in *B. pseudomallei*, where a trehalase (*treA*) mutant showed increased sensitivity to oxidative stress, even though the elevated trehalose levels in the mutant would be expected to protect against this stress [38]. In *S. mutanstreR* appears to play a role in resistance to oxidative stress including exposure to H_2_O_2_, but *treA* or *treB* mutants do not show a similar phenotype [21]. This suggests that genes outside of the trehalose operon, which are regulated by *treR*, are involved in resistance to oxidative stress, although the identity of the genes involved are not known [21]. In summary, the evidence is that trehalose rarely plays a role in protection against oxidative stress in bacteria.

**Table 1.** Trehalose biosynthesis or degradation mutants with altered resistance to abiotic stresses.

Bacterial species	Mutant tested	Phenotype(s) reported
***Trehalose biosynthesis***		
*A. baumannii*	*otsB*	Mutant grew more slowly than wild type at 40°C at 50 mM NaCl. Wild type, but not mutant, grew at 45°C ^[33]^.
*E. coli*	*otsA*	mutant survived poorly at 4°C for 5 days (10% survival) compared to wild type (25%) or *otsBA* over-expressing strain (>50%)^[12]^
*E. coli*	*otsA otsB*	Reduced survival of mutant at 55°C compared to wild type^[34]^
*Ralstonia solanacearum*	*otsA* or *treY, treS, otsA*	Both mutants show increased susceptibility to osmotic stress (0.15 M NaCl or 15% polyethylene glycol 400). No difference in resistance to high (47°C) or low (20°C) temperature stress, dessication, oxidative stress^[47]^.
*Rhizobium etli*	*otsA*	Reduced growth of mutant, compared to wild type, at 35°C. Reduced (3-fold) survival of mutant, compared to wild type, following dessication^[36]^
*Rhizobium leguminosarum*	*otsATreY*	Wild type cells survived 100-fold more than mutant cells after dessication for 6 weeks^[11]^
*Salmonella enterica*	*otsA*	Reduced survival of mutant, compared to wild type, at 50°C^[35]^
*Xanthomonascitri*	*otsA*	Reduced survival of mutant, compared to wild type, when exposed to osmotic stress (300 mM NaCl) or oxidative stress (30 mM H_2_O_2_)^[42]^
**Trehalose degradation**		
*B. pseudomallei*	*treA*	Increased survival of mutant, compared to wild type, at 4°C or 65°C. Decreased survival when exposed to 40 mM tert-butyl hydroperoxide. There were no reported differences in susceptibilities to pH4, H_2_O_2_ or O_2_^−^^[38]^.
*Listeria monocytogenes*	*treA*	Compared to wild type the mutant showed increased survival at 52°C and in 20% NaCl and dessication. There were no reported differences in susceptibilities to 18% ethanol, 0.1% H_2_O_2_, pH 3.5 or 4°C ^[37]^

The power of trehalose to protect against abiotic stresses lies in the ability of this sugar to limit damage to biological molecules, and this is believed to be a consequence of several mechanisms acting together [1,2]. Trehalose is chemically stable and the 1,1 glycosidic linkage provides conformational rigidity rendering its resistance to hydrolysis. However, this is not the key feature which distinguishes this sugar from other disaccharides. A trehalose glass can form around biological molecules and would be stable at high temperatures and when desiccated. This glass can form at typical environmental or mammalian body temperatures and could physically entrap biological molecules [43]. At these temperatures, trehalose can also exist in different crystalline forms that entrap water [43]. The ability to transition between these different forms whilst retaining its structural properties, especially at the temperatures of biological systems, might be an important feature that distinguishes this sugar from other disaccharides [43]. The protective potential of trehalose also appears to lie with the ability of the molecule to preferentially form bonds with water molecules i.e. the bond between trehalose and water is stronger than the water:water bond. Whilst other disaccharides are also able to displace water, the hydration number for trehalose is higher. The conformational stability of trehalose provides a stable hydration shell and allows the molecule to both make and break water bonds [2]. Therefore, trehalose can displace water molecules that would normally be hydrogen bonded to the biological molecule and a hydration shell is organized around the trehalose molecules in the solvation layer [2,43].

Finally, it is believed that trehalose can protect proteins and unsaturated fatty acids from oxidative damage and the efficiency of this also distinguishes trehalose from other disaccharides [2]. Trehalose is able to interact with fatty acids possessing *cis* double bonds, preventing oxidative damage and the formation of hydroperoxides [2]. It is proposed that similar interactions of trehalose with *cis* double bonds of the aromatic side chains of some amino acids could prevent protein aggregation [2]. Protein stabilization may also be associated with the role of trehalose in limiting Nε-lysine acetylation of proteins which would otherwise result in increased protein hydrophobicity and aggregation [44]. Nε-lysine acetylation of proteins may be a consequence of carbon overflow within the bacterial cell. Therefore, in this case trehalose can function both as a chemical chaperone andas a metabolite that limits carbon overflow in biological systems [44].

The ability of trehalose to protect bacterial cells from a range of stresses might explain the role of this sugar in the survival of bacteria both inside and outside of a host. This seems to be especially true for bacterial pathogens of plants. For example, in *Rhizobium leguminosarum* [11], *Sinorhizobium meliloti* [45] and *Bradyrhizobium japonicum* [46], the ability of trehalose to protect against abiotic stresses has been associated with enhanced colonization of plant root nodules, possibly by providing increased resistance to osmotic stress in plant tissues [46]. *Ralstonia solanacearum* is a major cause of bacterial wilt disease and mutants with compromised trehalose biosynthesis show reduced abilities to colonize tomato plants and to cause disease in soil-soak infection studies. These mutants also show reduced resistance to osmotic stress, but not to other *in vitro*stresses. This might reflect the role of trehalose in promoting survival in the xylem sap where osmotic strength might vary depending on sugar levels [47]. Interestingly, during infection, the elevated level of trehalose found in the xylem is believed to be of plant rather than bacterial origin. This might also contribute to osmotic stress encountered by the bacteria.

### Colonization of the host

There are a number of examples of trehalose playing a role in the colonization of plants by bacterial pathogens. As highlighted above, in some cases this may reflect the role of trehalose in protecting bacteria against the stresses encountered in plant tissues. The authors have previously found that trehalase plays a role in virulence of *B. pseudomallei* [38]. Deletion of the *treA* gene had a marked effect on growth of this pathogen in cell cultures and virulence in *Galleria mellonella* and in mice [38]. As highlighted above, the *treA* mutant showed a modest increase in susceptibility to oxidative stress, though whether this alone is sufficient to explain the marked reduction in virulence is uncertain.

But, there is also evidence that trehalose enabling colonization of the host by mechanisms other than protecting against stresses. A potential role of the periplasmic trehalase (*treA*) in virulence was an unexpected finding from a study to identify transposon mutants in extra-intestinal pathogenic *E. coli* (ExPEC) that were defective in binding to (and invasion of) non-phagocytic cells [48]. Moreover, the *treA* mutant showed reduced ability to colonize the bladder in a murine model of urinary tract infection, and the wild type phenotype was restored by complementation, indicating that polar effects were not responsible. This appears to be a consequence of the reduced abundance of type 1 fimbriae on the bacteria cell surface. Interestingly, this is not the first time that trehalose metabolism has been linked to virulence of ExPEC [49]. However, the molecular mechanisms that link trehalose to virulence in ExPEC are not clear.

### Pathogen growth

Trehalose is an important carbon source for many bacteria, and can support bacterial growth. The ability of bacteria to use trehalose as a carbon source could therefore explain the reported associations with virulence, by enabling bacteria growth *in vivo*. However, the utilization of trehalose as a carbon source is only likely to be beneficial to pathogens that grow at carbon-limited sites, for example outside of host tissues or cells.

*Clostridium difficile* causes an infection in the colon of humans and disease is largely a consequence of the production of toxins, which act on the gut. Collins *et al*. [50,51] recently showed that compared to most isolates of *C. difficile*, three phylogentically distinct ribotypes (RT027, RT028, and RT017) have acquired the ability to metabolize low concentrations of trehalose. These ribotypes are considered to include “hypervirulent” strains which are associated with the emergence, 20 or so years ago, of *C. difficile* as a leading cause of nosocomial infection. It has been suggested that the emergence of RT027 and RT078 isolates is linked to the introduction of trehalose as a food additive in N. America in the early years of the 21^st^ century. However, conversely trehalose had been approved for use as food supplement in Japan in the mid 1990’s, but this did not trigger the emergence of these ribotypes in this country.

Two distinct mechanisms explain the enhanced ability of hypervirulent strains to metabolize trehalose [50,51]. RT027 and RT017 strains have point mutations in the trehalose repressor and this mutations result in increased expression of the TreA trehalase, and in the case of RT027 strains over 500-fold increased sensitivity to trehalose is reported [50]. In contrast, RT078 strains had acquired a cluster of genes which enabled growth on low concentrations of trehalose [50]. Two experiments using mice with humanised microbiota and given trehalose orally, demonstrated the potential for trehalose to modulate disease. First, it was shown that the RT027 wild type strain was more virulent than a *treA* mutant [50]. Secondly, it was shown that compared to a water control, oral dosing with trehalose increased the risk of disease 3-fold [50]. Additionally, a recent study [52] has shown that feeding trehalose or lactotrehalose (a trehalase-resistant form of trehalose) did not promote growth of *C. difficile* CD027 in mice, and actually reduced the levels of markers of inflammation in gut issues. It is not clear why the results of this study are different to the previously reported effects of trehalose on the potentiation of *C. difficile* disease. However, different challenge doses were used in these studies, and this might influence the outcomes. Further work is required to understand the possible association of trehalose with *C. difficile* disease.

Two other important examples of the possible association between trehalose and growth of pathogens in the gastrointestinal tract have been reported. In extra-intestinal avian pathogenic *E. coli*, deletion of genes involved in the uptake of trehalose reduced the ability of the strain to cause experimental colibacillosis in poultry [49]. In *S. mutans*, the ability to use trehalose as a carbon source is believed to provide the bacterium with a competitive advantage in the oral cavity [21]. However, the interpretation of both of these studies may be complicated by the findings that TreR also appears to regulate genes outside of the *tre* operon in both pathogens [21,53,54]. For example, in *S. mutans* this may be a consequence of the TreR mediated downregulation of non-lantibiotic mutacins IV, V and VI [21], which act on other bacteria in the oral cavity.

A very different role for trehalose in pathogen growth has been reported in *Pseudomonas aeruginosa* infection of plants. Here, trehalose appears to enable the acquisition of nitrogen-containing nutrients promoting growth of the bacterium in *Arabidopsis thaliana* [55]. Consequently, *P. aeruginosa treYZ* and *treS* mutants show 50-fold reductions in growth in *A. thaliana* leaves, and the wild type phenotype could be recovered by co-dosing the mutant with trehalose, suggesting that altered plant signaling via T6P is not the reason for reduced virulence [55]. Rather, it is proposed that the osmotic gradient generated by the bacterial trehalose causes efflux of solutes, including nitrogen-rich compounds essential for pathogen growth, from plant cells into the extracellular spaces colonized by the bacteria [55]. The *treYZ* and *treS* mutants of *P. aeruginosa* which are highly attenuated in *A. thaliana* are not attenuated in mice, mice lacking the cystic fibrosis transmembrane conductance regulator protein, *Caenorhabditis elegans*, or *Drosophila melanogaster* [55] demonstrating the host-specific roles of *treYZ* and *treS* in disease.

### Trehalose and modulation of the host immune response

There are several well-documented examples of the modulation of host immunity in plants and in mammals, which is mediated by bacterial trehalose or trehalose derivatives. The decreased ability of an *otsA* mutant of *Xanthomonascitri* to colonize citrus leaves might reflect the decreased resistance of the mutant to salt and oxidative stresses. However, a paradoxical finding was that compared to wild type, infection of plants with this mutant resulted in the reduced expression of mitogen-activated protein-kinase 3 and -kinase 4, wrky30 transcription factor, lipoxygenase 2, and other pathogenesis related genes, which are all associated with host defense against infection responses [42]. The implication of this finding is that bacterial trehalose signals infection to the plant.

The role of TDM in virulence is best studied in *M. tuberculosis*. A remarkable property of TDM is that the biological activity of the molecule is dependent on its local environment. TDM on the cell surface protects bacterial cells from killing by macrophages because it inhibits phagosome fusion with lysosomes [56]. As a monolayer, for example, when TDM is presented in an oil-in-water emulsion, it is highly toxic [56] destroying cells in minutes upon contact [57]. Many of the features of tuberculosis, from pro-inflammatory cytokine production to cachexia and granuloma formation and even death, can be induced by TDM alone [58]. TDM may also play a key role in promoting the chronic inflammation associated with persistent infection [59] and administration of TDM can induce granulomas formation in mice [56]. TDM is recognized by multiple macrophage C-type lectin receptors including Mincle [57,60] and Dectin 3 [61]. Binding to Dectin 3 can induce Mincle expression via NFκB activation, potentiating the response to TDM [61]. TDM can also bind to the macrophage receptor with collagenous structure (MARCO) receptor and the bound TDM factor can then activate TLR2 signaling [58]. TDM binding results in the activation of macrophages and the production of inflammatory cytokines via the NFκB pathway [58,61] and nitric oxide generation [60]. The Mincle-dependent pathway is characterized by the promotion of Th1 and Th17 responses [62]. Mice dosed intra-tracheally with TDM show elevated levels of TNFα, IL-6, IL-10, IFNγ, IL-12 and IL-4 in lung homogenates and elevated levels of NO in broncho-alveolar lavage fluid [63]. However, the panel of cytokines produced may be dependent on the cell type. For example, TNFα is released from murine bone marrow dendritic cells, but not from human monocytes of dendritic cells exposed to TDM [62]. The role of the trehalose phospholipids diPT and PT in disease caused by *S. enterica* and *E. coli* has yet to be established, though the ability of these molecules to bind to Mincle suggests that they may contribute to virulence of these species.

### *Trehalose-6-phosphate accumulation limits the ability of* M. tuberculosis *to cause disease*

In *M. tuberculosis* and *Mycobacterium bovis* the *otsB* gene is essential for growth *in vitro* and this has been attributed to the intracellular accumulation of trehalose-6-phosphate in the mutant [64,65]. The *otsB* gene also appears to be required for the establishment of acute, but not chronic disease in mice [64]. There are no reports that the accumulation of trehalose-6-phosphate is lethal in any other bacteria species. Other than in *M. tuberculosis, otsB* does not appear in the database of essential genes for any other bacterial species. However, the accumulation of trehalose-6-phosphate is lethal in *C. elegans*and detrimental to the growth of fungi such as *Saccharomyces cerevisiae* [66]. In *S. cerevisiae* it is believed that the growth-limiting effects of trehalose-6-phosphate are a consequence of the inhibition of hexokinase II, which is involved in the first steps of glycolysis [66]. It is possible that a similar mechanism explains the toxicity of accumulated trehalose-6-phosphate in *M. tuberculosis* [65]. However, mapping of the transcriptome in trehalose-6-phosphate-stressed *M. tuberculosis* cells revealed a wide range of up-regulated genes, and a much smaller group of down-regulated genes. The extent of gene up-regulation in trehalose-6-phosphate-stressed cells might point toward global changes in the cell that increase the half-lives of mRNAs [64]. In support of this suggestion, the *vapB* antitoxin genes were upregulated in trehalose-6-phosphate-stressed cells, and this might serve to neutralize the activity of VapC RNAases [64].

### Trehalose metabolism pathways as a potential drug targets

Trehalose biosynthetic pathways play a major role in virulence in fungi and consequently there has been progress in targeting this pathway with new antifungal compounds. The trehalose biosynthetic pathways are attractive targets for antifungals because this pathway is absent in mammals [67,68]. For example, enzymes involved in trehalose-6-phosphate biosynthesis (TPS and TPP) play key roles in virulence traits, such as the ability to grow at 37°C, capsule production and melanin biosynthesis as well as in cell wall integrity, and the regulation of glycolysis [67,69]. The regulation of glycolysis by trehalose-6-phosphate appears to be mediated by the inhibition of hexokinase II [68].

In some species of fungitrehalase enzymes play roles in virulence, but they are generally considered to be less attractive targets for drug development. This is partly because of trehalase redundancy, and partly because trehalases are found in mammals [67–69]. Notwithstanding these concerns, validamycin is a trehalase inhibitor, and has activity against phytopathogenic fungi [67], because of its ability to inhibit the cell wall acid trehalase [70]. However, it is less active toward human pathogenic fungi [70].

In bacteria, and with some notable exceptions, trehalose metabolism is generally considered to be a less attractive target for antimicrobials. Many bacteria possess TPS and TPP enzymes. However, with the exception of the Mycobacteria, trehalose biosynthesis pathways do not appear to play an important role in disease.For example, an *otsA* mutant of *Salmonella enterica* Typhimurium was reported to show no reduction in virulence in mice [35] and global mutagenesis studies have confirmed that *otsA* and *otsB* do not play roles in virulence of *S. enterica* in humanized mice [71]. In a global mutagenesis study of *B. pseudomallei* neither *otsA* nor*otsB* were found to be required for growth or virulence [72,73]. However, interestingly the *B. pseudomallei* genome appears to possess 2 *otsA* genes (BPSL1044 and BPSL2410), and so the possibility that redundancy masks the roles of the individual genes cannot be excluded (only one of the *otsA* genes, BPSL2410, is organized into an operon with *otsB*). Therefore, the available evidence indicates that the trehalose biosynthesis pathway unlikely to be a good target for broad-spectrum antibacterial compounds. Notwithstanding these limitations, inhibitors of OtsB in bacteria have been reported [74]. OtsB requires metal ion cofactors, and EDTA is an inhibitor by chelating these ions. EDTA has no clinical utility as an antibacterial, but inhibitors with greater specificity have been reported. For example, trehalose 6 phosphate has been reported to be an inhibitor of the *P. aeruginosa* OtsB [75]. In *M. smegmatis* and *M. tuberculosis* the antibiotics diumycin and moenomycin can inhibit OtsB activity, but the doses required for 50% inhibition are high (50 and 100 µg/ml) and these drugs were less active toward the *M. tuberculosis* enzyme [76]. Recently, there has been some exciting work to devise inhibitors specific for OtsB of Mycobacteria which are based on substrate analogs. These results showed that 6-*N*-phosphonamide 6 (TNP) exhibited the highest inhibitory activity toward the *M. tuberculosis* OtsB [77].

There is some interest in developing inhibitors of the *C. difficile* TreA and drugs that target the *B. pseudomallei* TreA might have clinical utility. Validamycin A is not active against *C. difficile* TreA [78] but trehalose derivatives, such as epimers bearing hydroxyl groups at the 2- and 4-positions and also thiotrehalose derivatives, show potential as broader spectrum TreA inhibitors [78]. The value of any of these drugs for the treatment of specific bacterial diseases awaits investigation.

Apart from the trehalose metabolism pathways outlined above *Mycobacteria* additionally possess a number of specialized pathways with potential drug targets. These pathways in the *M. tuberculosis* have received attention because of the important roles that they play in survival and virulence [25]. Targets include the GlgE and maltokinase (Mak) enzymes in the GlgE pathway. A substrate analog of maltose-1-phosphate, maltose-C-phosphonate, shows promise as a lead compound [79]. The Pks13 enzyme, MmpL3 transporter, and Ag85 complex in the TMM/TDM biosynthesis pathways [25,80] are also attractive targets for anti-mycobacterial drugs. A new class of thiophene compounds have been shown to inhibit Pks13 and when combined isoniazid resulted in sterilizing activity [81]. However, possibly the most promising drug target for the *M. tuberculosis* is MmpL3. A range of MmpL3 inhibitors have been reported and SQ109, a 1,2-ethylene diamine, has been tested in clinical trials [25].

### Trehalose and vaccine manufacture

TDM is a potent activator of macrophages and activates the adaptive immune response as a consequence of binding to Mincle [60]. TDM is found in a number of experimental adjuvants including as a component of complete Freunds and Ribi adjuvants [60]. The toxicity of TDM makes it unsuitable for use in humans, but derivatives such as trehalose 6,6,’-dibehenate (TDB), are being investigated as less toxic analogs (Figure 2). TDB is still able to bind to Mincle and activate the NFκB pathway via Syk-Card9–Bcl10–Malt1 signaling, and cytokine production [62]. Like TDM, binding of TDB to Mincle, involves both glucose units and one acyl chain [82].

Monomolecular trehalose is well established as a stabilizing agent for a range of biological products, including vaccines. The applications of trehalose as a stabilizing agent for macromolecules is reviewed in a range of previous publications [43,83]. There are several reports of the addition of trehalose to stabilize live attenuated *Pasteurella multocida* [84], *E. coli* [85], *Francisella tularensis* [86], and *S. enterica* [87]. The exploitation of the endogenous trehalose biosynthetic pathway to stabilize live bacterial vaccines is less well documented. However, two reports suggest that the induction of trehalose biosynthesis can have detrimental effects on the infection competence of live attenuated *S. enterica*vaccines. In one study the loss of acid and bile salt tolerance was reported in bacteria subsequently stored at room temperature [88]. Others reported that growth in high salt medium induced trehalose in live attenuated *Salmonella*but resulted in the loss of invasion competence [89]. However, it is not clear whether the loss of virulence was a direct consequence of trehalose induction, or a consequence of the altered expression of other genes under high salt conditions. It is also not known whether infection competence would be reduced after immunization by other than the oral route.

## Discussion

Trehalose is a true multifunctional molecule, and numerous studies over the past two decades have revealed the roles of trehalose in fungal diseases. In fungi, trehalose acts as a stress protectant [67] though not an osmoprotectant [4] and yeasts are generally unable to grow using trehalose as a carbon source [4]. In fungi trehalose and trehalose-6-phosphate also play key roles in regulating metabolism and trehalose-6-phosphate is an important signaling molecule [67]. The role of trehalose in bacterial pathogens is less well understood, although we can draw on work with fungal pathogens to illuminate our understanding. In bacteria trehalose can serve as a carbon source and a stress protectant [4] but there is less evidence that trehalose or trehalose metabolites regulate metabolism or act as signaling molecules. Unlike fungi, trehalose is an important component of the cell wall in some bacterial species.

It is clear that trehalose metabolism can influence virulence in some bacterial species, from enabling growth to modulating the immune response to infection. However, there are a number of important questions that are yet to be addressed and in general the roles of trehalose metabolism and virulence are often pathogen-specific making it difficult to generalize. This observation suggests that broad spectrum antibacterial drugs or therapies, which target trehalose metabolism are unlikely. But pathogen-specific drug development is promising. The role of trehalose and trehalose derivatives in the regulation of metabolism and virulence is an area that merits further investigation.
